# Educational games for self-care of people with intestinal ostomies: a scoping review

**DOI:** 10.1590/0034-7167-2024-0662

**Published:** 2025-12-12

**Authors:** Camila Vicente, Lúcia Nazareth Amante, Lorena Barros da Silveira, Rizioléia Marina Pinheiro Pina, Huiana Cristine Lucca

**Affiliations:** I Universidade Federal de Santa Catarina, Empresa Brazileira de Serviços Hospitalares. Florianópolis, Santa Catarina, Brazil; II Universidade Federal de Santa Catarina. Florianópolis, Santa Catarina, Brazil; III Fundação Centro de Controle de Oncologia do Estado do Amazonas. Manaus, Amazonas, Brazil; IV Universidade Federal do Amazonas. Manaus, Amazonas, Brazil

**Keywords:** Nursing, Ostomy, Enterostomy, Self Care, Play and Playthings., Enfermería, Estomaterapia, Estomía, Autocuidado, Juego e Implementos de Juego.

## Abstract

**Objective:**

to describe scientific evidence of self-care games for people with intestinal stomas.

**Methods:**

a scoping review that included original, review, experimental, exploratory, descriptive, analytical, quasi-experimental, and experimental articles. Editorials, letters to the editor, abstracts, opinion pieces, duplicates, and studies involving animals or cadavers were excluded. The articles followed the following stages: export to Mendeley software; exclusion of duplicates; screening; test investigation; full-text reading; eligibility assessment; reference search; and article inclusion. Data extraction was conducted in accordance with JBI, which served as the basis for assessing methodological quality.

**Results:**

a total of 645 articles were identified, leaving two articles included. Article 1 consisted of a card game for adults, with probing questions about handling and maintenance. Article 2, a smartphone app called StoMakker, was for children and adolescents.

**Conclusions:**

we identified a lack of articles addressing the topic and the need to expand publications and develop these playful activities.

## RESUMEN

**Objetivo:**

describir la evidencia científica sobre juegos de autocuidado para personas con ostomías intestinales.

**Métodos:**

se realizó una revisión exploratoria que incluyó artículos originales, de revisión, de experiencia, exploratorios, descriptivos, analíticos, cuasiexperimentales y experimentales. Se excluyeron editoriales, cartas al editor, resúmenes, artículos de opinión, duplicados y estudios con animales o cadáveres. Los artículos siguieron los siguientes pasos: exportación a Mendeley; exclusión de duplicados; cribado; investigación de prueba; lectura del texto completo; evaluación de elegibilidad; búsqueda bibliográfica; e inclusión de artículos. La extracción de datos se realizó de acuerdo con el JBI, que sirvió de base para evaluar la calidad metodológica.

**Resultados:**

se identificaron 645 artículos, de los cuales dos fueron incluidos. El artículo 1 consistió en un juego de cartas para adultos, con preguntas de sondeo sobre su manejo y mantenimiento. El artículo 2, una aplicación para teléfonos inteligentes llamada StoMakker, estaba dirigido a niños y adolescentes.

**Conclusiones:**

se identificó una escasez de artículos que aborden el tema y la necesidad de ampliar las publicaciones y desarrollar estas actividades lúdicas.

## INTRODUCTION

An ostomy is the creation of an opening between an internal organ and the external environment through a surgical opening. The need for a stoma can arise for a variety of reasons, including planned surgery, elective surgery, or an emergency situation. A stoma can be temporary or permanent^(1,2)^.

People with intestinal ostomies and their families face changes and challenges that interfere with their daily and social life, as well as physical and psychological changes. This can be attributed to a lack of information, quality materials that provide comfort and safety, including adaptation of collection equipment, and fear of fecal and gas overflow, as well as the lack of adapted bathrooms^(3)^.

Studies have already shown a failure in communication, guidance and monitoring between the multidisciplinary team and people with intestinal stomas^(4,5)^, which leads to a deficiency in the self-care process^(4)^. These are the main cause of hospital readmission^(5)^, due to complications such as post-operative infections, prolapse or intestinal obstruction^(6-8)^.

Dorothea Orem’s Self-Care Deficit Theory states that every human being has basic skills and requirements to achieve self-care; however, some people need the assistance of a professional or caregiver/family member in this process to obtain the ability to carry out activities related to their own care^(9)^.

Most people with intestinal ostomies have not achieved Dorothea Orem’s assumptions^(10)^. From the perspective of this theory, nurses have the ability to help meet a person’s needs and highlight their strengths, taking into account that the action of teaching is a fundamental method to help the person become an agent of self-care^(9)^.

To this end, there are several educational technologies that can help professionals carry out this guidance and self-care, as they are relevant for people with intestinal ostomies in the recovery process, contributing to coping with the new life condition and redefining their self-image and self-concept, in overcoming fears and taboos^(11)^.

Among educational technologies, we have serious games, which consist of games composed of all the structural elements, but with a defined purpose, with a result or training that the creators wish to pass on to users, such as, in this case, a transformation of educational content into a game^(12)^.

A study shows that the development of these technologies helps in the learning of specific content and training, providing a less tiring, more enjoyable and motivating learning experience, compared to traditional learning methods, which do not include any technological or innovative elements^(13)^.

In this way, they are playful activities that have the capacity to assist in the learning process in an innovative, stimulating and fun manner^(14)^. Furthermore, they are recommended to encourage the acquisition of knowledge such as disease prevention, treatment, symptoms and procedures^(15)^, helping people with intestinal stoma to have their independence, autonomy and self-care from the hospital admission process to home^(16-17)^.

A serious game that addresses self-care aims to transform practices often seen as mandatory and tedious into more engaging, informative, and motivating experiences, promoting better adherence and, consequently, better health outcomes. In the specific context of people with ostomies, it can address issues such as insecurity regarding ostomy care, lack of clear and accessible information, fear of complications, and difficulty integrating self-care into daily routines.

Serious games for self-care in healthcare are still under-researched and under-explored in nursing practice. This study demonstrates the state-of-the-art in their development for people with ostomies in their self-care. The potential of serious games, with their intrinsic qualities and characteristics, offers a promising avenue for assisting in this regard, positioning the game as a tool that promotes self-care and offers significant support.

To develop these serious games, six stages are necessary^(18)^. The first stage, called the concept phase, consists of creating and documenting the game idea concisely and objectively, defined by a small team. This team works on identifying the market and assessing the resources available for project development, in addition to writing the conceptual proposal^(18)^.

To assist in market identification, which is essential for this initial phase, a scoping review was proposed. This type of study does not aim to find the best evidence, but rather to gather all existing evidence, identifying potential gaps in existing research on games that address self-care for people post-stoma surgery. A scoping review is a type of study that aims to map the literature in a given field of interest, covering broad topics, allowing for an understanding of the types of research, study designs, who conducted them, and the evidence produced^(19)^.

This manuscript consists of one of the stages of a master’s dissertation of the Graduate Program in Nursing Care Management, Professional Modality, *Universidade Federal de Santa Catarina* entitled “*Orientação de enfermagem para o autocuidado da pessoa com estomia intestinal: uso do serious games*”, registered in the Certificate of Presentation for Ethical Appreciation 81427524.2.0000.0121, which is in the process of finalization and will be defended in a panel scheduled for September 2025.

## OBJECTIVE

To describe scientific evidence on games that address self-care for people in the post-operative period of intestinal ostomies.

## METHODS

### Ethical aspects

Ethical assessment and approval were waived for this study, as it was a review, and all content used is in the public domain, not involving participation in studies with human beings.

### Study design

This is a scoping review study, following the scoping review protocol, the guide model for preparation by Girondi and Soldera^(20)^, the JBI^(21)^ theoretical and methodological framework and the writing based on the Preferred Reporting Items for Systematic reviews and Meta-Analyses extension for Scoping Reviews of 2020 (PRISMA-ScR).

The review included the stages recommended by JBI^(21)^, namely: 1) Develop the research question; 2) Determine inclusion and exclusion criteria; 3) Develop a protocol (including history, objectives, inclusion/exclusion criteria, search strategy, data extraction and synthesis methods, and assessment of methodological quality); 4) Register the protocol; 5) Develop and implement a search strategy; 6) Select studies by analyzing titles and abstracts; 7) Extract data from the included studies; 8) Assess methodological quality; 9) Synthesize the data (grouping, summary, and presentation of results) according to PRISMA-ScR 2020.

From these stages, the guiding research question was structured using the acronyms P (population), I (intervention), Co (context), with P representing person with intestinal ostomy, I, educational games, and Co, self-care. The following question emerged from the scoping review: what self-care games have been developed for people in the postoperative period of intestinal ostomies?

### Search period, strategy and source

The review period ran from March to July 2024. The selected search platforms were the National Library of Medicine (PubMed) and the Virtual Health Library (VHL). The electronic databases used were: Medical Literature Analysis and Retrieval System Online (MEDLINE); *Literatura Latino-Americana de Ciências da Saúde* (LILACS); *Banco de Dados em Enfermagem* (BDENF); Scientific Electronic Library Online (SciELO); Scopus; Cumulative Index of Nursing and Allied Health Literature (CINAHL); Web of Science (WoS); Excerpta Medica Database (EMBASE); Educational Resources Information Centre (ERIC); Cochrane Library; and the first ten pages of Google Scholar.

The subjects for selecting descriptors and keywords were based on “games” and “intestinal ostomy”, as shown in Chart 1.

**Chart 1:** Keywords and descriptors used in the search strategy. Florianópolis, SC, Brazil, 2024

Language	Subject 1	Subject 2
Portuguese	*Jogos de Vídeo; Videojogos; Videojogo; Jogos; Jogo; Jogos Recreativos; Gamificação; Aplicativos Móveis*	*Estomia; Ostomia; Ileostomia; Colostomia*
Spanish	*Juegos de Video; Juegos; Juego; Juegos Recreacionales; Gamificación; Aplicaciones Móviles; Aplicación móvil*	*Estomia; Ostomia; Ileostomia; Colostomia*
English	Video Games [MeSH]; Video Games; Videogame; Videogames; Game; Games; Games, Recreational [MeSH]; Recreational Games; Gamification; Mobile Applications [MeSH]; Mobile Applications; App; Apps.	Ostomy [MeSH]; Ostomy; Ostomies; Ileostomy [MeSH]; Ileostomy; Ileostomies; Colostomy [MeSH]; Colostomy; Colostomies

The search strategy was developed with the assistance of a librarian who is an expert in this type of research, using Health Sciences Descriptors for databases in Portuguese. For LILACS, Spanish descriptors were used. For databases in English, Medical Subject Headings (MeSH) descriptors were used. Along with the descriptors, the Boolean terms AND and OR were used to compose the search keys. The search strategy is available in Chart 2.

**Chart 2:** Search strategies. Florianópolis, SC, Brazil, 2024

Database	Search strategy
PubMed/MEDLINE	(“Video Games” [MeSH] OR “Video Games” OR “videogame” OR “videogames” OR “Game” OR “Games” OR “Games, Recreational” [MeSH] OR “Recreational Games” OR “Gamification” OR “Mobile Applications” [MeSH] OR “Mobile Applications” OR “App” OR “Apps”) AND (“Ostomy” [MeSH] OR “Ostomy” OR “Ostomies” OR “Ileostomy” [MeSH] OR “Ileostomy” OR “Ileostomies” OR “Colostomy” [MeSH] OR “Colostomy” OR “Colostomies”)
LILACS/BDENF, SciELO	(“Video Games” OR “videogame” OR “videogames” OR “Game” OR “Games” OR “Recreational Games” OR “Gamification” OR “*Jogos de Vídeo*” OR “*Videojogos*” OR “*Videojogo*” OR “*Jogos*” OR “*Jogo*” OR “*Jogos Recreativos*” OR “*Gamificação*” OR “*Juegos de Video*” OR “*Juegos*” OR “*Juego*” OR “*Juegos Recreacionales*” OR “*gamificación*” OR “*Aplicativos Móveis*” OR “*Aplicaciones Móviles*” OR “*aplicación móvil*” OR “Mobile Applications” OR “App” OR “Apps”) AND (“Ostomy” OR “Ostomies” OR “Ileostomy” OR “Ileostomies” OR “Colostomy” OR “Colostomies” OR “*Estomia*” OR “*Ostomia*” OR “*Ileostomia*” OR “*Colostomia*”)
EMBASE, CINAHL, Cochrane Library, Scopus, WoS, ERIC	(“Video Games” OR “videogame” OR “videogames” OR “Game” OR “Games” OR “Recreational Games” OR “Gamification” OR “Mobile Applications” OR “App” OR “Apps”) AND (“Ostomy” OR “Ostomies” OR “Ileostomy” OR “Ileostomies” OR “Colostomy” OR “Colostomies”)
Google Scholar (English)	(“Video Games” OR “videogame” OR “videogames” OR “Game” OR “Games” OR “Recreational Games” OR “Gamification” OR “Mobile Applications” OR “App” OR “Apps”) AND (“Ostomy” OR “Ostomies” OR “Ileostomy” OR “Ileostomies” OR “Colostomy” OR “Colostomies”)
Google Scholar (Spanish and Portuguese)	(“Video Games” OR “videogame” OR “videogames” OR “Game” OR “Games” OR “*Videojogos*” OR “*Videojogo*” OR “*Jogos*” OR “*Jogo*” OR “*Gamificação*” OR “*Juegos*” OR “*Juego*” OR “*gamificación*” OR “App”) AND (“*Estomia*” OR “*Ostomia*” OR “*Ileostomia*” OR “*Colostomia*”)

*Legend: PubMed - National Library of Medicine; MEDLINE - Medical Literature Analysis and Retrieval System Online; LILACS - Literatura Latino-Americana de Ciências da Saúde; BDENF - Banco de Dados em Enfermagem; SciELO - Scientific Electronic Library Online; CINAHL - Cumulative Index of Nursing and Allied Health Literature; WoS - Web of Science; ERIC - Educational Resources Information Centre.*

### Inclusion and exclusion criteria

Original articles available online through access via the Federated Academic Community, review articles (integrative, narrative and systematic), experience reports, exploratory, descriptive, analytical, quasi-experimental, experimental studies, published in Portuguese, English or Spanish, without a time frame, were included.

Editorials, letters to the editor, proceedings of scientific events (abstracts) or conferences, opinion articles, duplicate publications and studies whose study population was not human beings were excluded.

### Study protocol

After elaboration and external validity of the protocol, it was registered in the Open Science Framework (OSF), under DOI registration: 10.17605/OSF.IO/FRH6P and available for access at: <https://archive.org/details/osf-registrations-frh6p-v1>.

The first stage took place on March 10, 2024, and was conducted by the lead author through a search for studies using the aforementioned database search strategy. The identified records were exported to Mendeley, a bibliographic reference manager, to identify duplicates and remove them. After the software removed duplicates, the remaining records were shared by creating a group within the manager for peer review.

The first reading, known as screening, consisted of reviewing the titles and abstracts of each reference and determining whether the study was eligible based on the inclusion criteria. This stage occurred between March 20 and June 20, 2024. Subsequently, on July 12, 2024, a peer-to-peer consensus meeting was held to compare the results. During a consensus meeting, a small number of records explicitly addressing the topic in their abstracts and titles were identified. Therefore, it was decided to include articles that discussed apps and other technological tools but did not clearly state their approach (whether they were games or not). These studies underwent a cursory reading, searching for terms related to game(s) to determine eligibility, which was unclear in the first reading. This stage was then identified as a test investigation.

Between July 12 and July 17, 2024, the selected articles were read in full, and this stage was identified as full-text reading, analyzing them according to the inclusion criteria. A new consensus meeting was held on July 17, 2024, during which all reasons for exclusion were explained. At this point, it was decided to also search the references of the final selected articles, reading the title, abstract, and full text, as needed to determine whether the inclusion criteria were met.

### Analysis of results

The remaining articles in the final review sample underwent a data extraction process according to the research objective, in line with the JBI, which based the assessment of methodological quality and helped compile the data into a synoptic table in Microsoft Excel, including: author(s); title; year of publication; database; study objective; methodological design (method: quantitative/qualitative/mixed; nature of the research: study design); study location (participants or population/sample); main results/outcomes; conclusion: responding to the objective.

The data were critically analyzed, identifying problems and highlighting obvious gaps, and described narratively. During the discussion, the results were synthesized, reflecting the research question and objectives, to suggest implications for future research, programs, and/or public policies.

## RESULTS

**Figure 1:**
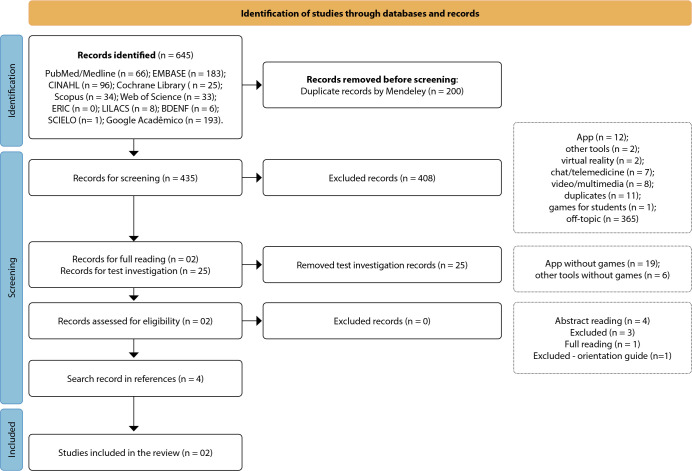
Identification of studies through databases and records according to the Preferred Reporting Items for Systematic reviews and Meta-Analyses extension for Scoping Reviews. Florianópolis, SC, Brazil, 2024

The results of all stages were organized and demonstrated according to PRISMA-ScR and can be identified in Figure 1.

The selected studies comprised Chart 3 presented below:

**Chart 3:** Results of studies included in the scoping review. Florianópolis, SC, Brazil, 2024

Title or reference(s) (^*^)	Year	Design/number of patients	Interventions	Outcomes
*Atividade lúdica no processo educacional ao paciente estomizado* ^(22)^	2021	Descriptive, exploratory and qualitative n=7	Identify: 1) Patient knowledge about ostomy; 2) Learning linked to the educational game; and 3) Difficulties in the game.	It was found that, after playing the card game, the patient was able to understand what a stoma entails. The playful activity implemented in this study provided important guidance and contributed to improving intestinal stoma care. The playful activity proved to be a useful tool that facilitated patients’ learning and understanding process, highlighting the importance of self-care not only for patients’ physical well-being but also for their psychological well-being.
StoMakker: improving the Quality of Life of Children Receiving an Ileostomy, Colostomy or Continent Urostomy by Offering Access to a Peer Support Platform, Age Dependent Information Provision and Games in a Smartphone Application^(23)^	2022	Multicenter, quantitative, randomized controlled clinical trial n = no information	The primary outcome measures identify the change from baseline in participants’ health-related quality of life on the seven-item PROMIS Pediatric Global Health scale (PGH-7) age-specific questionnaire at six months post-surgery. Secondary outcome measures include: 1) Change from baseline in participants’ anxiety on the eight-item, age-specific PROMIS Pediatric v2.0 Anxiety and Depressive Symptoms questionnaire at six months post-surgery; 2) Change from baseline in participants’ social functioning on the age-specific PROMIS Pediatric Item Bank, Short Form, Peer Relationships, at six months post-surgery; 3) Occurrence of any surgery-related complications, as specified by the Clavien-Dindo classification, within six months of follow-up. The scale consists of several grades (grades I, II, IIIa, IIIb, IVa, IVb, and V).	

In the first article^(22)^, the game consisted of a card game for adults, with probing questions about the correct handling and maintenance, developed by researchers, including surgical experts. The game had 30 playing cards, divided into three groups. The first was called “inquisitive” because it contained probing cards that presented questions regarding the digestive ostomy. The second, called “lucky”, included correct answers to the probing messages. Finally, the last group, called “inaccurate”, contained cards that presented erroneous arguments regarding the question in the written message. For each probing card presented to patients, a lucky and an inaccurate message were offered, and patients were free to choose the card that best suited the questions.

In the second article^(23)^, the smartphone app called StoMakker, for children and adolescents, contained games, called serious games, based on the user’s age, which ranged from 6 to 18 years, but did not provide details on how the game worked or its benefits. Furthermore, this game is not aimed solely at intestinal ostomies, but includes other types of ostomies. This article is merely a record of ongoing research that has not yet had its final version published.

## DISCUSSION

A gap was identified in the production of studies focused on the field of serious games associated with the teaching-learning process with encouraging self-care aimed at people with intestinal ostomy, as two studies addressing the topic were identified.

The first study developed an educational activity aimed at people with intestinal ostomies through an analog card game^(22)^. The other^(23)^ brought a serious game in application mode, one for adults and one for children, respectively.

Through their research, they identified that the use of games for health guidance proved to be an enriching and effective tool for encouraging self-care among people with intestinal ostomies, by providing guidance that involves knowledge about the ostomy and prevention of complications^(22)^.

The use of this playful activity favored teaching and learning, as it intentionally sparked the attention and curiosity of a person with an intestinal ostomy. In this scenario, nursing must act to facilitate and encourage this educational process^(22)^.

Furthermore, the StoMakker app sought to identify improvements in quality of life for children living with some type of ostomy, determining whether app use leads to knowledge retention, satisfaction, competence, and self-care skills. The research suggested that a gaming app could improve the quality of life for ostomy patients by providing personalized support, facilitating contact with like-minded individuals, and offering a reliable and accessible information base, ultimately improving postoperative quality of life^(23)^.

The study also revealed that, prior to using the games, there was a lack of knowledge about nutritional and dietary factors, as well as a lack of health guidance and encouragement for self-care for intestinal ostomies. Through the game, they gained an understanding of what an intestinal ostomy is, the necessary care, and knowledge of the human body to prevent complications^(22)^. This fact is in line with the study on the StoMakker application, which, despite not yet having published the final results, predicts the influence of the game on the quality of life, anxiety, social functioning and occurrence of health-related complications of participants using the application up to six months after surgery^(23)^.

Difficulties in using the game are related to cultural factors, the language used in the cards, and educational level, issues that must be taken into account when developing an educational game for this audience. To this end, it was suggested to combine a written tool with pictures to reduce comprehension biases^(22)^.

The tool highlights the importance of health education for guiding and encouraging self-care, as well as its role in facilitating patients’ learning and understanding process, bringing benefits not only to patients’ physical status, but also psychological status, as it brings up topics that will be part of the daily lives of patients with intestinal ostomies, demonstrating to patients how to interact correctly with their bodies^(22)^.

Although the topic of “self-care and people with intestinal ostomies” is a broad topic in nursing studies, its association with game development demonstrates that it remains an open field with little involvement from nurses. Furthermore, it represents an area for scientific advancement, entrepreneurship, and the consolidation of nursing science, with the application of serious game concepts in postoperative education.

Despite the scarcity, the identified articles provide scientific evidence on games that address self-care for people in the post-operative period of intestinal ostomies related to the benefits of using games as a playful activity for self-care, understanding what a stoma is, guidance on necessary ostomy care, and aspects focused on quality of life.

### Study limitations

The research highlights the use of educational games as a technological tool for teaching and learning self-care. We suggest expanding the research to other types of playful activities that, through the use of technology, aid in the process of educating and developing self-care.

### Contributions to health, nursing or public policy

This study served to identify the market, contributing to the first stage of developing an educational game called the concept phase^(18)^ by identifying gaps in existing research on games that address self-care for people in the post-operative period of intestinal ostomies.

Based on this, and taking into account that nursing has a great emphasis on caring for people with intestinal ostomies and has the potential to apply the concepts of serious games to develop playful activities that develop self-care, this study allowed the writing of a conceptual proposal for an educational game in board format for use in hospital surgical clinics, in order to encourage and educate self-care for patients with intestinal ostomies and their families.

## CONCLUSIONS

The scoping review identified the lack of scientific evidence on games that address self-care for people in the postoperative period of intestinal ostomies, achieving the study’s objective.

Although nursing plays an essential role in health education, the development and validity of educational tools, and the understanding and care of people with intestinal ostomies, there is a lack of research focusing on the use of educational games for this topic. This creates a gap in the dissemination of scientific knowledge among professionals and, consequently, in the teaching and learning process of these patients.

This highlights the need to expand research on this topic and develop recreational activities to develop self-care for this population so that future studies can be published that address the creation and validity of educational games specifically aimed at people with intestinal ostomies.

It is worth highlighting that nursing, as a major player in caring for this population, should be encouraged to develop research applicable in this scenario to contribute both to the field and to the scientific and population community.

## Data Availability

As it is a scoping review, it is also registered in the OSF, under DOI registration: 10.17605/OSF.IO/FRH6P and available for access at: <https://archive.org/details/osf-registrations-frh6p-v1>.
