# Supplementary material for: Educational games for self-care of people with intestinal ostomies: a scoping review
Source: Rev Bras Enferm. 2025 Dec 12;78(Suppl 4):e20240662. doi: 10.1590/0034-7167-2024-0662 (PMC12707841; doi:10.1590/0034-7167-2024-0662)
Supplement: Translation

# Translation: Jogos educativos para o autocuidado da pessoa com estomia intestinal: revisão de escopo

**DOI:** 10.1590/0034-7167-2024-0662pt

**Authors:** Camila Vicente, Lúcia Nazareth Amante, Lorena Barros da Silveira, Rizioléia Marina Pinheiro Pina, Huiana Cristine Lucca

**Affiliations:** I Universidade Federal de Santa Catarina, Empresa Brasileira de Serviços Hospitalares. Florianópolis, Santa Catarina, Brasil; II Universidade Federal de Santa Catarina. Florianópolis, Santa Catarina, Brasil; III Fundação Centro de Controle de Oncologia do Estado do Amazonas. Manaus, Amazonas, Brasil; IV Universidade Federal do Amazonas. Manaus, Amazonas, Brasil

**Keywords:** Enfermagem, Estomaterapia, Estomia, Autocuidado, Jogos e Brinquedos.

## Abstract

**Objetivo:**

descrever as evidências científicas dos jogos sobre autocuidado das pessoas com estomias intestinais.

**Métodos:**

revisão de escopo que incluiu artigos originais, de revisão, de experiência, exploratórios, descritivos, analíticos, quase-experimentais e experimentais. Excluíram-se editoriais, cartas ao editor, resumos, opinião, duplicadas e estudos com animais ou cadáveres. Os artigos seguiram as etapas: exportação para o *software* Mendeley; exclusão dos duplicados; triagem; investigação teste; leitura na íntegra; avaliação da elegibilidade; busca nas referências; inclusão de artigos. A extração de dados ocorreu em consonância ao JBI, que baseou a avaliação da qualidade metodológica.

**Resultados:**

identificaram-se 645 artigos, restando dois artigos incluídos. O artigo 1 consistia em jogo de cartas, para adulto, com perguntas indagadoras sobre manuseio e manutenção. O artigo 2, aplicativo de *smartphone* denominado *StoMakker*, era para crianças e adolescentes.

**Conclusões:**

identificaram-se a incipiência de artigos que abordem a temática e a necessidade da expansão de publicações e elaboração dessas atividades lúdicas.

## INTRODUÇÃO

A estomia consiste na criação de abertura entre o órgão interno e o meio externo por meio de orifício confeccionado durante procedimento cirúrgico. A necessidade de sua confecção pode ser gerada por diferentes causas, incluindo as planejadas cirurgicamente, de forma eletiva, ou em decorrência de uma situação de emergência, podendo ser temporárias ou definitivas^(1,2)^.

A pessoa com estomia intestinal e seus familiares enfrentam mudanças e desafios que interferem no seu convívio cotidiano e social, assim como alterações no contexto físico e psicológico. Este contexto pode ser atribuído à falta de informação, material de qualidade que traga conforto e segurança, incluindo adaptação do equipamento coletor e receio de extravasamento das fezes e gases, assim como ausência de banheiros adaptados^(3)^.

Estudos já têm evidenciado uma falha na comunicação, orientação e acompanhamento entre a equipe multidisciplinar e as pessoas com estomias intestinais^(4,5)^, o que leva a uma deficiência no processo do autocuidado^(4)^. Essas são a maior causa de readmissão hospitalar^(5)^, por complicações como infecções de pós-operatórias, prolapso ou obstrução intestinal^(6-8)^.

A Teoria do Déficit de Autocuidado de Enfermagem de Dorothea Orem aborda que todo ser humano possui competências e requisitos básicos para alcançar o autocuidado; contudo, algumas pessoas precisam do auxílio de um profissional ou cuidador/familiar nesse processo para obtenção da capacidade de realizar as atividades referentes ao seu próprio cuidado^(9)^.

A maioria das pessoas com estomias intestinais não alcançou os pressupostos de Dorothea Orem^(10)^. Na perspectiva dessa teoria, o enfermeiro tem capacidade para ajudar a suprir as necessidades da pessoa e evidenciar suas potências, devendo levar em consideração que a ação de ensinar é um método fundamental para ajudar a pessoa a se tornar um agente do autocuidado^(9)^.

Para isso, existem diversas tecnologias educacionais que podem ajudar os profissionais a realizar essa orientação e autocuidado, pois são relevantes para pessoas com estomias intestinais no processo de recuperação, contribuindo para enfrentamento da nova condição de vida e ressignificação de sua autoimagem e autoconceito, na superação dos medos e tabus^(11)^.

Entre as tecnologias educacionais, temos os *serious game*, que consistem em jogos compostos por todos os elementos estruturais, mas tendo um propósito definido, com resultado ou capacitação que os criadores desejam passar para o usuário, como, no caso, uma transformação de conteúdo educativo em um jogo^(12)^.

Estudo mostra que o desenvolvimento dessas tecnologias auxilia na aprendizagem de conteúdos e treinamentos específicos, proporcionando uma experiência de aprendizagem menos cansativa, mais prazerosa e motivadora, em comparação aos métodos tradicionais de aprendizagem, que não incluem nenhum elemento tecnológico ou inovador^(13)^.

Dessa forma, são atividades lúdicas que apresentam capacidade de auxiliar no processo de aprendizagem de forma inovadora, estimulante e divertida^(14)^. Além disso, são indicadas para o estímulo da obtenção de conhecimentos como prevenção de doenças, tratamento, sintomas e procedimentos^(15)^, contribuindo para que a pessoa com estomia intestinal assuma a sua independência, autonomia e autocuidado desde o processo de internação hospitalar até o domicílio^(16-17)^.

*Serious game* que aborda o autocuidado visa transformar práticas muitas vezes vistas como obrigatórias e maçantes em experiências mais engajadoras, informativas e motivadoras, promovendo uma melhor adesão e, consequentemente, melhores resultados de saúde. No contexto específico de pessoas estomizadas, pode abordar problemas como a insegurança em relação aos cuidados com a estomia, a falta de informação clara e acessível, o medo de complicações e a dificuldade em integrar o autocuidado à rotina diária.

Os *serious* games na área da saúde para o autocuidado ainda são pouco abordados e explorados na prática dos profissionais de enfermagem. A realização deste estudo demonstra o estado da arte no seu desenvolvimento para pessoas estomizadas em seu autocuidado. O potencial do *serious game*, com suas qualidades e características intrínsecas, oferece uma via promissora para auxiliar nesse aspecto, posicionando o jogo como uma ferramenta que promove o autocuidado, oferecendo um suporte significativo.

Para o desenvolvimento desse *serious games*, é necessária a realização de seis etapas^(18)^. A primeira etapa, denominada de fase de conceito, consiste no momento da criação e do registro da ideia do jogo, de modo sucinto e objetivo, definido por uma equipe reduzida. Esta equipe trabalha na identificação do mercado e avaliação dos recursos disponíveis para o desenvolvimento do projeto, além de escrever sua proposta conceitual^(18)^.

Para auxiliar na identificação do mercado, essencial para essa fase inicial, foi proposta revisão de escopo, pois esse tipo de estudo não visa buscar a melhor evidência, mas reunir todos os tipos de evidência que já foram produzidas, identificando possíveis lacunas de pesquisas existentes sobre os jogos que abordam o autocuidado para pessoas em pós-operatório de estomias intestinais. A revisão de escopo é um tipo de estudo que visa realizar mapeamento da literatura em um determinado campo de interesse, com tópicos amplos, permitindo compreender os tipos de pesquisas, os desenhos de estudos, por quem foram realizadas e as evidências produzidas^(19)^.

Este manuscrito consiste em uma das etapas da dissertação de mestrado do Programa de Pós-Graduação em Gestão do Cuidado em Enfermagem, Modalidade Profissional, da Universidade Federal de Santa Catarina intitulado “Orientação de enfermagem para o autocuidado da pessoa com estomia intestinal: uso do *serious games”*, registrado no Certificado de Apresentação para Apreciação Ética n^o^ 81427524.2.0000.0121, que está em processo de finalização e será defendido em banca programada para setembro de 2025.

## OBJETIVO

Descrever as evidências científicas sobre os jogos que abordam o autocuidado para pessoas em pós-operatório de estomias intestinais.

## MÉTODOS

### Aspectos éticos

A avaliação e aprovação ética foram dispensadas para este estudo, por se tratar de revisão, e todo conteúdo utilizado possui domínio público, não envolvendo participação de estudos com seres humanos.

### Desenho do estudo

Estudo de revisão de escopo, seguindo o protocolo de *scoping review,* o modelo guia para elaboração de Girondi e Soldera^(20)^, o referencial teórico e metodológico do JBI^(21)^ e a redação baseada no *Preferred Reporting Items for Systematic reviews and Meta-Analyses extension for Scoping Reviews* de 2020 (PRISMA-ScR).

A revisão contemplou as etapas recomendadas por JBI^(21)^, a saber: 1) Elaborar a pergunta da pesquisa; 2) Determinar critérios de inclusão e exclusão; 3) Desenvolver um protocolo (incluindo histórico, objetivos, critérios de inclusão/exclusão, estratégia de busca, métodos de extração e síntese de dados, e avaliação da qualidade metodológica); 4) Registrar protocolo; 5) Desenvolver e implementar estratégia de busca; 6) Selecionar os estudos analisando títulos e resumos; 7) Extrair dados dos estudos incluídos; 8) Avaliar a qualidade metodológica; 9) Sintetizar os dados (agrupamento, resumo e apresentação dos resultados) conforme PRISMA-ScR 2020.

A partir dessas etapas, a questão norteadora da pesquisa foi estruturada utilizando o acrônimo P (população), I (intervenção), Co (contexto), sendo o P pessoa com estomia intestinal, I, jogos educativos, e Co, autocuidado. Emergiu a seguinte pergunta da revisão de escopo: quais são os jogos desenvolvidos sobre autocuidado para pessoas em pós-operatório de estomias intestinais?

### Período, estratégia e fonte de busca

O período da revisão ocorreu de março a julho de 2024. As plataformas de buscas selecionadas foram a *National Library of Medicine* (PubMed) e a Biblioteca Virtual em Saúde (BVS) . As bases de dados eletrônicas utilizadas foram: *Medical Literature Analysis and Retrieval System Online* (MEDLINE); Literatura Latino-Americana de Ciências da Saúde (LILACS); Banco de Dados em Enfermagem (BDENF); *Scientific Electronic Library Online* (SciELO); Scopus; *Cumulative Index of Nursing and Allied Health Literature* (CINAHL); *Web of Science* (WoS); *Excerpta Medica Database* (EMBASE); *Educational Resources Information Centre* (ERIC); *Cochrane Library;* e as dez primeiras páginas do *Google* Acadêmico.

Os assuntos para seleção dos descritores e palavras-chave foram baseados em “jogos” e “estomia intestinal”, como demonstrado no Quadro 1.

**Quadro 1:** Palavras-chave e descritores utilizados na estratégia de busca. Florianópolis, SC, Brasil, 2024

Idioma	Assunto 1	Assunto 2
Português	Jogos de Vídeo; Videojogos; Videojogo; Jogos; Jogo; Jogos Recreativos; Gamificação; Aplicativos Móveis	Estomia; Ostomia; Ileostomia; Colostomia
Espanhol	*Juegos de Video; Juegos; Juego; Juegos Recreacionales; Gamificación; Aplicaciones Móviles; Aplicación móvil*	*Estomia; Ostomia; Ileostomia; Colostomia*
Inglês	*Video Games* [MeSH]*; Video Games; Videogame; Videogames; Game; Games; Games, Recreational* [MeSH]; *Recreational Games; Gamification; Mobile Applications* [MeSH]; *Mobile Applications; App; Apps.*	*Ostomy* [MeSH]; *Ostomy; Ostomies; Ileostomy* [MeSH]; *Ileostomy; Ileostomies; Colostomy* [MeSH]; *Colostomy; Colostomies*

A estratégia de busca foi elaborada com assessoria de bibliotecária *expert* nesse tipo de pesquisa, a partir dos Descritores em Ciências da Saúde, para as bases em português. Para LILACS, utilizaram-se os descritores em espanhol. E para as bases de dados que utilizam o idioma inglês, utilizaram-se os descritores do *Medical Subject Headings* (MeSH). Junto aos descritores, foram empregados os termos booleanos *AND* e *OR* para compor as chaves de busca. A estratégia de busca está disponível no Quadro 2.

**Quadro 2:** Estratégias de Busca. Florianópolis, SC, Brasil, 2024

Base de dados	Estratégia de busca
PubMed/MEDLINE	(*“Video Games”* [MeSH] *OR “Video Games” OR “videogame” OR “videogames” OR “Game” OR “Games” OR “Games, Recreational”* [MeSH] *OR “Recreational Games” OR “Gamification” OR “Mobile Applications”* [MeSH] *OR “Mobile Applications” OR “App” OR “Apps”*) *AND* (*“Ostomy”* [MeSH] *OR “Ostomy” OR “Ostomies” OR “Ileostomy”* [MeSH] *OR “Ileostomy” OR “Ileostomies” OR “Colostomy”* [MeSH] *OR “Colostomy” OR “Colostomies”*)
LILACS/BDENF, SciELO	(*“Video Games” OR “videogame” OR “videogames” OR “Game” OR “Games” OR “Recreational Games” OR “Gamification” OR* “Jogos de Vídeo” *OR* “Videojogos” *OR* “Videojogo” *OR* “Jogos” *OR* “Jogo” *OR* “Jogos Recreativos” *OR* “Gamificação” *OR “Juegos de Video” OR “Juegos” OR “Juego” OR “Juegos Recreacionales” OR “gamificación” OR “Aplicativos Móveis” OR “Aplicaciones Móviles” OR “aplicación móvil” OR “Mobile Applications” OR “App” OR “Apps”*) *AND* (*“Ostomy” OR “Ostomies” OR “Ileostomy” OR “Ileostomies” OR “Colostomy” OR “Colostomies” OR* “Estomia” *OR* “Ostomia” *OR* “Ileostomia” *OR* “Colostomia”)
EMBASE, CINAHL, *Cochrane Library,* Scopus, WoS, ERIC	(*“Video Games” OR “videogame” OR “videogames” OR “Game” OR “Games” OR “Recreational Games” OR “Gamification” OR “Mobile Applications” OR “App” OR “Apps”*) *AND* (*“Ostomy” OR “Ostomies” OR “Ileostomy” OR “Ileostomies” OR “Colostomy” OR “Colostomies”*)
*Google* Acadêmico (inglês)	(*“Video Games” OR “videogame” OR “videogames” OR “Game” OR “Games” OR “Recreational Games” OR “Gamification” OR “Mobile Applications” OR “App” OR “Apps”*) *AND* (*“Ostomy” OR “Ostomies” OR “Ileostomy” OR “Ileostomies” OR “Colostomy” OR “Colostomies”*)
*Google* Acadêmico (espanhol e português)	(“*Video Games” OR “videogame” OR “videogames” OR “Game” OR “Games” OR* “Videojogos” *OR* “Videojogo” *OR* “Jogos” *OR* “Jogo” *OR* “Gamificação” *OR “Juegos” OR “Juego” OR “gamificación” OR “App”*) *AND* (“Estomia” *OR* “Ostomia” *OR* “Ileostomia” *OR* “Colostomia”)

*Legenda: PubMed - National Library of Medicine; MEDLINE - Medical Literature Analysis and Retrieval System Online; LILACS - Literatura Latino-Americana de Ciências da Saúde; BDENF - Banco de Dados em Enfermagem; SciELO - Scientific Electronic Library Online; CINAHL - Cumulative Index of Nursing and Allied Health Literature; WoS - Web of Science; ERIC - Educational Resources Information Centre.*

### Critérios de inclusão e exclusão

Artigos originais disponíveis *online* a partir do acesso via Comunidade Acadêmica Federada, artigos de revisão (integrativa, narrativa e sistemática), relatos de experiência, estudos exploratórios, descritivos, analíticos, quase-experimentais, experimentais, publicados nos idiomas português, inglês ou espanhol, sem recorte temporal, foram incluídos.

Editoriais, cartas ao editor, anais de eventos científicos (resumos) ou conferências, artigos de opinião, publicações duplicadas e estudos cuja população estudada não seja em seres humanos foram excluídos.

### Protocolo do estudo

Após elaboração e validação externa do protocolo, o mesmo foi registrado no *Open Science Framework* (OSF), sob registro DOI: 10.17605/OSF.IO/FRH6P e disponível para acesso em: <https://archive.org/details/osf-registrations-frh6p-v1>.

A primeira etapa ocorreu no dia 10 de março de 2024, e foi realizada pela autora principal, por meio da busca de estudos, utilizando a estratégia de busca nas bases de dados previamente citadas. Os registros identificados foram exportados para o *software* Mendeley, que é um gerenciador de referências bibliográficas, a fim de apontar materiais repetidos e excluí-los. Após a exclusão das duplicatas por meio do *software,* os registros restantes foram compartilhados por meio da criação de um grupo dentro do próprio gerenciador para leitura em pares.

A primeira leitura, identificada como triagem, consistiu em percorrer os títulos e resumos de cada referência e decidir se o estudo era elegível conforme os critérios para inclusão. Essa etapa ocorreu entre os dias 20 de março e 20 de junho de 2024. Após esse momento, no dia 12 de julho de 2024, foi realizada reunião de consenso entre os pares a fim de comparar os resultados. Durante a reunião de consenso, identificou-se um número baixo de registros que abordavam explicitamente a temática nos resumos e títulos. Com isso, optou-se por incluir os artigos que falavam de aplicativos e outras ferramentas tecnológicas, mas que não deixavam claro o tipo de abordagem (podendo ser jogos ou não). Esses estudos passaram por uma leitura flutuante, em busca de termos relacionados a jogo(s), a fim de determinar a elegibilidade, que não estava clara na primeira leitura, e essa etapa ficou identificada como investigação teste.

Entre os dias 12 de julho e 17 de julho de 2024, os artigos selecionados foram lidos na íntegra, e essa etapa foi identificada como leitura na íntegra, analisando-os conforme os critérios de inclusão. Uma nova reunião de consenso foi realizada no dia 17 de julho de 2024, durante a qual todas as razões para exclusão dos registros foram explicitadas. Nesse momento, optou-se por realizar também uma busca nas referências dos artigos finais selecionados, por meio da leitura do título, resumo e íntegra, conforme necessidade para compreensão se os critérios de inclusão estariam envolvidos.

### Análise dos resultados

Os artigos restantes na amostra final da revisão passaram por um processo de extração de dados de acordo com o objeto da pesquisa, em consonância com o JBI, que baseou a avaliação da qualidade metodológica e auxiliou a compilação dos dados em um quadro sinóptico no *Microsoft Excel*, incluindo: autor(es); título; ano de publicação; base de dados; objetivo do estudo; desenho metodológico (método: quantitativo/qualitativo/misto; natureza da pesquisa: tipo de estudo); local do estudo (participantes ou população/amostra); principais resultados/desfechos; conclusão: responder ao objetivo.

Os dados foram comentados criticamente, identificando os problemas e apontando as lacunas evidentes, e foram descritos de forma narrativa. Na discussão, os resultados foram sintetizados, resgatando a pergunta de pesquisa e os objetivos, para sugerir as implicações para futuras pesquisas, programas e/ou políticas públicas.

## RESULTADOS

Os resultados de todas as etapas foram organizados e demonstrados de acordo com o PRISMA-ScR, podendo ser identificados na Figura 1.

**Figura 1:** Identificação dos estudos por meio de bancos de dados e registros conforme *Preferred Reporting Items for Systematic reviews and Meta-Analyses extension for Scoping Reviews*. Florianópolis, SC, Brasil, 2024

Os estudos selecionados compuseram o Quadro 3, apresentado a seguir.

**Quadro 3:** Resultados dos estudos incluídos na revisão de escopo. Florianópolis, SC, Brasil, 2024

Título ou referência (s) (^*^)	Ano	Delineamento/número de pacientes	Intervenções	Desfechos
Atividade lúdica no processo educacional ao paciente estomizado^(22)^	2021	Descritivo, exploratório e qualitativo n=7	Identificar: 1) Conhecimento do paciente sobre estomia; 2) Aprendizagem vinculada ao jogo educativo; e 3) Dificuldades no jogo.	Foi possível identificar que, após a aplicação do jogo de cartas, o paciente conseguiu compreender o que seria uma estomia. A atividade lúdica aplicada neste estudo foi capaz de fornecer orientações importantes e contribuir na melhoria dos cuidados com a estomia intestinal. A atividade lúdica aplicada se mostrou uma ferramenta apta e facilitadora do processo de aprendizagem e compreensão do paciente, que ressalta a importância que o autocuidado pode trazer não só para o estado físico do paciente, mas também para o seu psicológico.
*StoMakker: improving the Quality of Life of Children Receiving an Ileostomy, Colostomy or Continent Urostomy by Offering Access to a Peer Support Platform, Age Dependent Information Provision and Games in a Smartphone Application* ^(23)^	2022	Ensaio clínico randomizado controlado multicêntrico e quantitativo n= sem informação	Nas medidas de resultados primários, identifica-se a mudança em relação à linha de base na qualidade de vida relacionada à saúde dos participantes no questionário específico para idade da PROMIS *Pediatric Global Health scale* (PGH-7), de sete itens, seis meses após a cirurgia. As medidas de resultado secundários incluem: 1) Mudança em relação à linha de base na ansiedade dos participantes no questionário *PROMIS pediatric v2.0 Anxiety and Depressive Symptoms*, de oito itens, específico para a idade, em seis meses de acompanhamento; 2) Mudança em relação à linha de base no funcionamento social dos participantes no banco de itens pediátricos do PROMIS específico para a idade, formulário curto, relacionamentos entre pares, seis meses após a cirurgia;3) Ocorrência de quaisquer complicações relacionadas à cirurgia, conforme especificado pela classificação de Clavien-Dindo, dentro de seis meses de acompanhamento. A escala consiste em vários graus (graus I, II, IIIa, IIIb, IVa, IVb e V).	

No primeiro artigo^(22)^, o jogo consistia em um jogo de cartas, para o público adulto, com perguntas indagadoras que questionavam a forma correta de manuseio e manutenção, elaborado pelos pesquisadores, incluindo especialistas na área cirúrgica. O jogo possui 30 cartas, estilo baralho, que foram divididas em três grupos. O primeiro recebeu o nome de indagador, por conter cartas indagadoras, que apresentavam questionamentos com relação à estomia digestiva. O segundo, nomeado de sorte, incluía respostas corretas das mensagens indagadoras. Por fim, o último grupo, intitulado de inexato, continha as fichas que apresentavam argumentação errônea em relação ao questionamento da mensagem escrita. Para cada peça indagadora que foi apresentada ao paciente, uma mensagem de sorte e uma inexata foram ofertadas, ficando ao seu critério a escolha da carta que melhor se adequa às perguntas.

Já no segundo artigo^(23)^, o aplicativo via *smartphone* denominado *StoMakker*, para crianças e adolescentes, continha jogos, denominados *serious games*, de acordo com a idade do usuário, que variava de 6 a 18 anos, mas não traziam detalhes de como o jogo funcionava e seus benefícios. Além disso, esse jogo não é voltado apenas para estomias intestinais, incluindo outros tipos de estomias. Esse artigo é apenas o registro de pesquisa em andamento que ainda não teve sua versão final publicada.

## DISCUSSÃO

Identificou-se uma lacuna na produção de estudos voltados ao campo de *serious games* associado ao processo de ensino-aprendizagem com o estímulo do autocuidado voltado à pessoa com estomia intestinal, pois houve a identificação de duas pesquisas abordando a temática.

O primeiro estudo elaborou uma atividade educacional voltada para pessoas com estomia intestinal por meio de jogo de cartas analógicas^(22)^. Já o outro^(23)^ trouxe um *serious game* na modalidade de aplicativo, sendo um jogo para adultos e um para crianças, respectivamente.

Por meio de sua pesquisa, identificaram que o uso dos jogos para orientação em saúde demonstrou ser uma ferramenta enriquecedora e efetiva para estimular o autocuidado das pessoas com estomia intestinal, por meio do fornecimento de orientações que envolvem o conhecimento sobre a estomia e prevenção de complicações^(22)^.

O uso dessa atividade lúdica favoreceu o ensino e aprendizagem, pois despertou a atenção e curiosidade de forma intencional da pessoa com estomia intestinal. Nesse cenário, a enfermagem deve atuar de maneira facilitadora e incentivadora desse processo educativo^(22)^.

Além disso, no aplicativo *StoMakker*, buscou-se identificar o aumento da qualidade de vida em crianças que convivem com algum tipo de estomia, verificando se o uso do aplicativo retém o conhecimento, traz satisfação, competência e capacidade para o autocuidado. Houve a perspectiva de que um aplicativo de jogos possa aprimorar a qualidade de vida dos pacientes com estomia ao fornecer suporte personalizado, facilitar contato com indivíduos na mesma condição e oferecer uma base de informações confiável e acessível, o que acaba por melhorar a qualidade de vida no pós-operatório^(23)^.

O estudo permitiu identificar também que, antes do uso dos jogos, identificou-se ausência de conhecimento sobre fatores nutricionais e alimentares, assim como omissão de orientações em saúde e estímulo do autocuidado com a estomia intestinal. A partir do jogo, houve a compreensão do que é uma estomia intestinal, os cuidados necessários e o conhecimento do corpo humano para prevenção de complicações^(22)^. Tal fato vai ao encontro do estudo sobre o aplicativo *StoMakker*, que apesar de ainda não possuir publicação dos resultados finais, prevê a influência do jogo na qualidade de vida, ansiedade, funcionamento social e ocorrência de complicações relacionadas à saúde dos participantes no uso do aplicativo até seis meses após a cirurgia^(23)^.

As dificuldades com o uso do jogo estão relacionadas a fatores culturais, linguagem utilizada nas cartas e nível de escolaridade, questões que devem ser levadas em consideração ao elaborar um jogo educativo para esse público. Para isso, houve a sugestão de associar uma ferramenta escrita com gravuras para diminuir os vieses de compreensão^(22)^.

A ferramenta destaca a importância da educação em saúde para orientação e estímulo do autocuidado, assim como o seu papel de facilitadora do processo de aprendizagem e compreensão do paciente, trazendo benefícios não só para o estado físico, mas também para o estado psicológico dos pacientes, pois traz assuntos que irão fazer parte do cotidiano dos pacientes com estomias intestinais, demonstrando aos pacientes como interagir da forma correta com o seu corpo^(22)^.

Apesar de a temática “autocuidado e pessoa com estomia intestinal” ser um assunto com abrangência nos estudos na área da enfermagem, a sua associação com o desenvolvimento de jogos demonstra ser um campo aberto com pouca inserção do enfermeiro. Além disso, é uma área para avanço científico, empreendedorismo e consolidação da ciência da enfermagem, com a aplicação dos conceitos de *serious games* no campo da educação pós-operatória.

Apesar da escassez, os artigos identificados trazem evidências científicas sobre jogos que abordam o autocuidado para pessoas em pós-operatório de estomias intestinais relacionadas aos benefícios da utilização de jogos como atividade lúdica para o autocuidado, compreensão do que é uma estomia, orientações sobre os cuidados necessários com a estomia e aspectos voltados à qualidade de vida.

### Limitações do estudo

Destaca-se a realização apenas com a abordagem dos jogos educativos como ferramenta tecnológica de ensino e aprendizagem do autocuidado. Sugere-se a expansão da pesquisa para outros tipos de atividades lúdicas que, por meio do uso da tecnologia, auxiliem no processo de educação e desenvolvimento do autocuidado.

### Contribuições para a área da saúde, enfermagem ou políticas públicas

Este estudo serviu para identificação do mercado, contribuindo para a primeira etapa para a elaboração de jogo educativo denominada de fase de conceito^(18)^, por meio da identificação das lacunas de pesquisas existentes sobre os jogos que abordam o autocuidado para pessoas em pós-operatório de estomias intestinais.

A partir disso, e levando em consideração que a enfermagem possui grande destaque nos cuidados com a pessoa com estomia intestinal e potencial na aplicação dos conceitos de *serious games* para elaboração de atividades lúdicas que desenvolvam o autocuidado, este estudo permitiu a escrita da proposta conceitual de jogo educativo no formato tabuleiro para uso nas clínicas cirúrgicas hospitalares, a fim de incentivar e educar o autocuidado ao paciente com estomia intestinal e seus familiares.

## CONCLUSÕES

A revisão de escopo identificou a incipiência de evidências científicas sobre os jogos que abordam o autocuidado para pessoas em pós-operatório de estomias intestinais, alcançando o objetivo do estudo.

Apesar de a enfermagem desempenhar um papel essencial na educação em saúde, na construção e validação de ferramentas educativas, e no conhecimento e cuidado com a pessoa com estomia intestinal, nota-se uma escassez de pesquisas voltadas para o uso dos jogos educativos para a temática. Isso gera uma lacuna na disseminação de conhecimentos científicos entre os profissionais e, consequentemente, para uso no processo de ensino-aprendizagem desses pacientes.

Evidencia-se, assim, a necessidade de expansão de pesquisas sobre essa temática e elaboração de atividades lúdicas para o desenvolvimento do autocuidado desse público, para que possam ser publicados futuros estudos que abordem a criação e validação dos jogos educativos voltadas especificamente a pessoas com estomias intestinais.

Vale ressaltar que a enfermagem, como grande destaque nos cuidados com esta população, deve ser incentivada a desenvolver pesquisas aplicáveis neste cenário, para contribuir tanto para a área quanto para a comunidade científica e populacional.

## Data Availability

Por se tratar de revisão de escopo, também está registrado no OSF, sob registro DOI: 10.17605/OSF.IO/FRH6P e disponível para acesso: <https://archive.org/details/osf-registrations-frh6p-v1>.
